# Data-driven rapid assessment of bridge responses under mixed traffic with restricted overloaded vehicles using machine learning

**DOI:** 10.1371/journal.pone.0355597

**Published:** 2026-08-11

**Authors:** Baoguo Luo, Qiankun Li, Junfeng Wang, Guohua Wang, Jianzhong Wu, Zigang Wang, Chongchong Zhan

**Affiliations:** 1 Guangdong Provincial Expressway Development Co.Ltd., Guangzhou, Guangdong, China; 2 Guangdong Communication Planning & Design Institute Group Co., Ltd, Guangzhou, Guangdong, China; 3 College of Civil Engineering, Xi’an University of Architecture and Technology, Xi’an, Shaanxi, China; 4 Highway College, Chang’an University, Xi’an, Shaanxi, China; National University of Singapore, SINGAPORE

## Abstract

For infrastructure and regional economic development, restricted overload vehicles (ROVs) are an indispensable part. These vehicles are typically of large size and heavy weight, and their impact on bridge structures and other infrastructure is much greater than that caused by the load of ordinary vehicles. Authorization from the competent transportation authority shall be obtained prior to road operation of such vehicles. With the increasing volume of ROVs on highway networks, the safety assessment of bridges under mixed traffic conditions has become a critical yet challenging task. In such scenarios, ROVs coexist with ordinary vehicles under open traffic conditions, which is not adequately addressed by traditional assessment methods. Simplified single-beam models fail to capture spatial load coupling effects, while the high-fidelity finite element simulations are too computationally expensive for rapid and batch processing. To bridge this gap, this study proposes a machine learning-based rapid prediction framework for estimating load effect amplification factors in mixed traffic. A high-fidelity grillage finite element model is established to systematically simulate bridge responses under various combinations of vehicle parameters, bridge types, and standard design loads. An influence surface-based moving load analysis method is employed to efficiently generate a comprehensive database covering over 2,000 ROVs across 17 typical small- and medium-span bridges. Using this database, seven machine learning algorithms are trained to predict the amplification factor from ROVs and bridge features. Results show that tree-based models, especially XGBoost, achieve high prediction accuracy with *R*^2^ > 0.98 for most load and response types. SHAP analysis reveals that while axle load has a limited effect under current load limits, the number of trailer axles is critical across all response types, with axle spacing and flexural rigidity governing positive moment and shear amplification, whereas span length and total bridge length predominantly control negative moment amplification. The proposed method allows for rapid estimation of mixed traffic effects by amplifying the single-vehicle response from a simplified model, offering a practical and efficient tool to support intelligent and real-time permit decision-making in bridge safety management.

## 1. Introduction

Restricted overload transport vehicles (hereinafter referred to as ROVs) are defined as vehicles that exceed standard weight limits and carry non-divisible, oversized cargo. Authorization from the competent transportation authority shall be obtained prior to road operation of such vehicles; otherwise, they shall be deemed illegal over-limit vehicles. As a crucial carrier for critical equipment in major infrastructure projects, the highway traffic safety of ROVs (especially during bridge crossings) has attracted significant attention in both academic and engineering circles [1,2]. According to the statistical survey conducted by the Ministry of Transport of the People’s Republic of China, the number of completed permits for ROVs in China reached 1.96 million in 2023, and the number is increasing year by year. This growing trend urgently calls for more intelligent and efficient management of ROV road operations. Particularly against the backdrop of rapid Intelligent Transportation System (ITS) [3–5], it is imperative to establish an efficient and safe assessment method for ROV bridge crossings, which is of great significance for ensuring traffic safety and advancing ITS.

ROVs typically have a total vehicle-cargo weight far exceeding design standards, coupled with distinctive geometric dimensions, and may induce significant local effects and global responses to bridge structures [6–8].To ensure safety, domestic and international specifications generally mandate temporary traffic control during ROVs’ bridge crossings to enforce a single-vehicle passage scenario. However, with growing traffic demand, the conflict between prolonged, large-scale traffic control and socioeconomic efficiency has become increasingly acute. Therefore, it is necessary to explore the mode in which these ROVs can share roads and bridges with ordinary vehicles, in order to maximize the efficiency of road transportation. However, this open-traffic scenario creates a fundamental discrepancy between actual bridge loading and the single-vehicle assumption in codes and conventional methods, posing a major challenge for bridge safety management [9,10].

In terms of assessment content, the safety assessment of highway bridges subjected to ROVs is largely analogous to that under regular vehicle loads. Professional assessment authorities typically conduct a comparison between the bridge load effects induced by ROVs and the existing ultimate bearing capacity of bridges, in accordance with current bridge design specifications and bridge bearing capacity evaluation standards [11], to determine whether ROVs meet the passage requirements. Therefore, the scientific and efficient calculation of bridge load effects under ROV action is an indispensable component of safety assessment. Regarding the calculation methods for load effects during ROVs’ bridge crossings, research progress has followed an evolutionary trajectory: from simplification to sophistication, from static to dynamic analysis, and from single-vehicle scenarios to the preliminary incorporation of environmental impacts [12].Early studies primarily employed the approach of simplifying the bridge as a single beam combined with the transverse distribution coefficient to assess responses under standard vehicle fleets or single-vehicle loads. This method features simplicity in calculation, acting as the foundation for initial engineering assessments, and remains one of the predominantly utilized calculation approaches by ROV passage assessment authorities [13].To more precisely simulate the spatial mechanical behaviors of bridges, especially under mixed traffic conditions involving ROVs and regular vehicles, the grillage method is one of the most extensively employed simulation techniques. By discretizing the bridge deck system into a grid of longitudinal and transverse beams, it can effectively capture the transverse distribution of loads and has been widely adopted for the safety assessment of newly constructed and existing bridges [14]. Subsequently, more sophisticated models (e.g., shell finite element and solid finite element models) have been employed to address complex phenomena such as vehicle-bridge coupling vibration and local stress concentration [15],and these models are typically restricted to large bridges of high significance or with complex local configurations [16]. With respect to load models, studies initially utilized pure static load models to approximate the dynamic effects of vehicles [17], and subsequently integrated additional influencing parameters such as road surface roughness and vehicle speed variations [18].Sophisticated simulation methods incur high computational costs and low assessment efficiency. Consequently, for most ROV assessments, simplified methods using a single beam with a transverse distribution coefficient remain predominant. In contrast, the grillage method, solid finite element method, and other sophisticated approaches are utilized less commonly.

However, notable limitations become apparent when evaluating mixed traffic scenarios [19]. First, regarding the objects of analysis, the overwhelming majority of studies remain focused on “single-vehicle” or “homogeneous fleet” bridge-crossing scenarios. A small number of studies involving the parallel travel of ROVs and regular vehicles are often confined to individual specific cases: they simplify regular vehicle loads to standard lane loads, compute the corresponding load effects, and then superimpose them with those induced by ROVs. This superposition approach neglects the load interaction effects during the parallel movement of ROVs and regular vehicles, resulting in relatively conservative internal force and load effect results. Second, in terms of methodological efficiency and applicability, to accurately capture the spatial load effects of mixed traffic, the only currently reliable approach is to develop refined spatial finite element models for bridges (e.g., grillage models or shell models) and perform dynamic loading simulations [15]. This process demands a high level of professional expertise in modeling, is computationally time-consuming, and entails substantial costs for a single analysis. Confronted with millions of ROV transportation permit applications and an extensive inventory of existing bridges in China each year, this refined simulation-based assessment framework struggles to meet the requirements for rapid and batch approval decisions. Although some studies have attempted to simplify the process by defining “influence line superposition areas” or employing “equivalent loads”, the accuracy and generalizability of these approaches remain difficult to ensure under highly variable conditions of vehicle parameters, bridge types, and traffic combinations. Thus, a practical dilemma exists: simplified single-beam models cannot account for spatial load coupling in mixed traffic, potentially leading to unsafe underestimation, while high-fidelity spatial models are too inefficient for large-scale assessments. Conversely, if a precise spatial model is utilized for full-scale simulations, the low efficiency renders it incapable of handling large-scale assessment tasks, making it difficult to support the urgent demand for real-time or quasi-real-time intelligent assessment amid the digital transformation of industry management.

To address the aforementioned issues, this study proposes a machine learning-based rapid assessment method for predicting the mixed traffic effect amplification factor. First, via refined finite element simulations, a comprehensive load effect database is systematically constructed to cover combinations of different ROV parameters, bridge types, and regular vehicle load levels. Subsequently, machine learning algorithms are employed to develop a prediction model that maps vehicle and bridge characteristics to the load effect amplification factor. In practical applications, the response of a single ROV crossing a bridge can first be computed using the single-beam method integrated with the transverse distribution coefficient, and then corrected using the factor predicted by this model, thereby enabling efficient acquisition of the approximate load effect under mixed traffic. This provides a feasible pathway for realizing the rapidness and intelligence of bridge safety assessment for ROV transportation.

## 2. Overall method and engineering application framework

**Fig 1:**
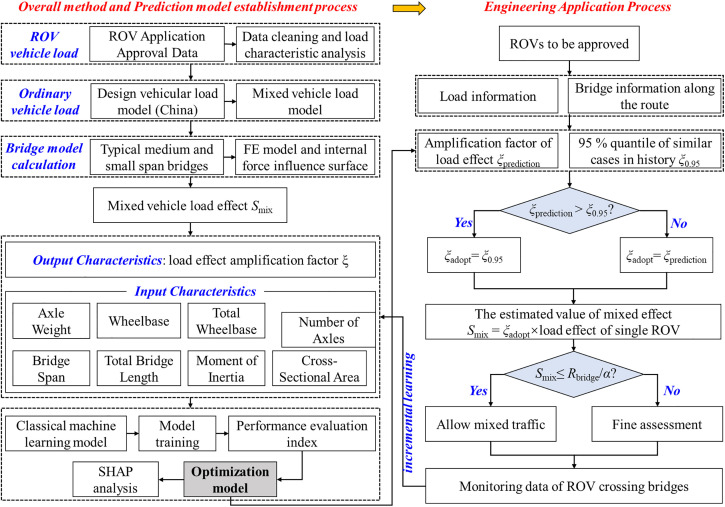
Overall method and engineering application framework.

To address the growing administrative burden caused by the surging number of ROV permit applications, as well as the safety assessment challenges arising from mixed-traffic scenarios in which ROVs share bridges with ordinary vehicles, this paper proposes a data-driven rapid evaluation method for estimating the bridge load effects induced by mixed traffic consisting of ROVs and ordinary vehicles (as shown in Fig 1). The overall framework of this study comprises the following components:

(1) Actual ROV permit application data from a provincial region in China are first subjected to data cleaning and statistical analysis of vehicle load parameters. This step identifies the typical distributions of axle weights and wheelbases, which then serve as the input source for the subsequent computation of load-effect datasets.
(2) The standard design vehicle load models specified in Chinese bridge design codes are adopted to represent ordinary vehicle loads. These are combined with ROV loads to form mixed vehicle load models; the detailed composition rules are presented in Section 4.3.
(3) Seventeen representative short- and medium-span bridges are selected, covering various span combinations, cross-sectional types, and material properties. Grillage finite element (FE) models are established using ANSYS software. For computational efficiency, the influence surfaces corresponding to the critical internal force responses are extracted, enabling batch loading of diverse mixed-vehicle load cases.
(4) The mixed-traffic load effect amplification factor is defined as the ratio of the load effect induced by mixed traffic to that induced by a single ROV crossing alone. Typical ROV loading parameters and bridge characteristics are selected as input features to develop a predictive model for this amplification factor. Seven classical machine learning algorithms are compared in terms of predictive performance. A total of nine predictive models are established, corresponding to different response types (e.g., bending moment, shear force) and ordinary-vehicle load levels. The SHAP (SHapley Additive exPlanations) method is further employed to analyze the influence patterns of the various feature parameters.

Through the above analytical framework, a generalized prediction model for the mixed-traffic load effect amplification factor is obtained. When a pending ROV permit application is submitted, technical personnel only need to input the prescribed vehicle parameters and combine them with the bridge parameters along the route. The model then predicts the corresponding amplification factor. Multiplying this factor by the load effect of the single ROV crossing the bridge—which can be conveniently obtained via a simplified single-beam method with transverse distribution coefficients—yields the estimated mixed-traffic load effect.

It should be noted that the proposed prediction model incorporates simplifications regarding ordinary traffic loading and bridge condition states. Moreover, under certain specific conditions, the model may produce slightly underestimated predictions, albeit with low probability. To ensure safety in engineering practice, the following procedural steps are recommended to guarantee the validity and safety redundancy of the load-effect estimation:

(1) The predicted amplification factor for the target ROV shall be taken as the maximum between the current prediction and the 95th percentile value of historical records for similar cases. ROVs with comparable axle-load and wheelbase distributions are automatically classified into the same category within the system, and their prediction results are stored in the historical database.
(2) The load-effect amplification factor is multiplied by the single-ROV crossing load effect to obtain the mixed-traffic effect estimate, denoted as *S*_mix_. If *S*_mix_ ≤ *R*_bridge_/*α*, where *R*_bridge_ is the bridge resistance and α is the safety margin coefficient (typically taken as 1.2–1.5), the permit is approved; otherwise, a refined evaluation is triggered. It is emphasized that *R*_bridge_ here refers to the modified resistance after accounting for the deterioration of the bridge technical condition. Based on our research team’s previous findings, the deterioration coefficients for bridges classified as Grades 1, 2, and 3 under Chinese bridge technical condition evaluation standard are taken as 1.1, 1.0, and 0.8, respectively.
(3) Finally, field monitoring data collected after vehicle passage are fed back into the model to enable online incremental learning, thereby continuously improving prediction accuracy and mitigating deviations under extreme scenarios.

## 3. Analysis of load characteristics of ROVs

**Fig 2:**
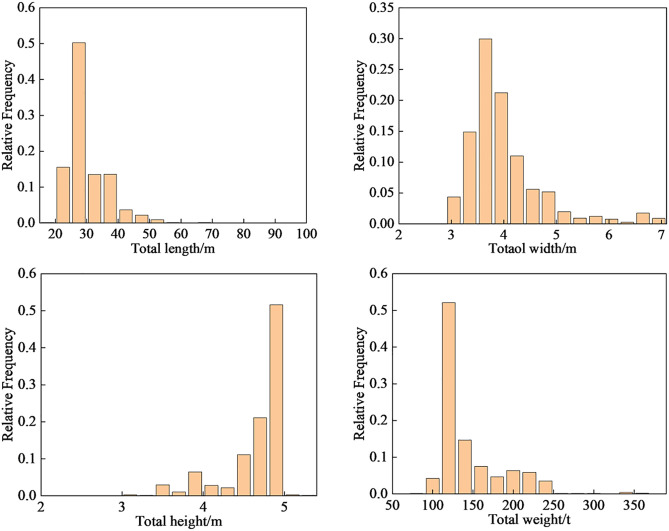
Statistical distributions of total weight and overall dimensions of ROVs.

The load characteristics of ROVs constitute the fundamental basis for the mechanical effects on bridge structures. To accurately evaluate the safety of ROVs during bridge crossing, it is essential to obtain the actual geometric and weight parameters. This study draws on the permit data of over 2,000 Class I ROVs from Guangdong Province, one of China’s most economically developed provinces with the highest expressway mileage and ROV traffic volume nationwide, collected over a single year. The dataset covers a wide range of total weights (80–300 t), axle configurations from 4 to 18 axles, and diverse wheelbase arrangements, thus representing the main ROV types commonly encountered on Chinese highways. The statistical distributions of the total weight and overall dimensions of ROVs (with cargo) are presented in Fig 2. As illustrated in Fig 2, the total length of ROVs is concentrated in the range of 20–40 m, the total width in 3–5 m, the total height in 3–5 m, and the total weight in 100–250 t.

**Fig 3:**
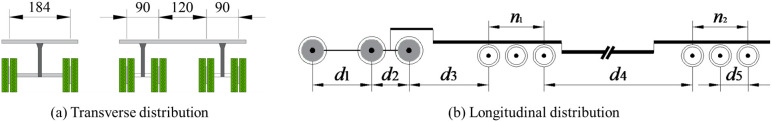
Distributions of transverse wheel spacing and longitudinal wheelbase of ROVs.

To further analyze the load distribution of ROVs, the transverse wheel spacing and longitudinal wheelbase of these vehicles are examined in detail. A typical ROV comprises a tractor and a trailer, featuring two transverse wheel arrangement configurations: the single-line configuration is typically employed for the steering axle of the tractor, while the double-line configuration is predominantly used for the load-carrying axle of the trailer. The most prevalent dimensional specification is presented in Fig 3(a): the transverse wheel spacing of the single-line type is generally 1.84 m, and that of the double-line type is typically 0.9 + 1.2 + 0.9 m. These identical dimensions are adopted for subsequent loading analyses [18].

Based on the position and function of the wheels, the longitudinal spacings of ROVs are categorized into five components: *d*1 (steering axle wheelbase), *d*2 (drive axle wheelbase), *d*3 (tractor-trailer spacing), *d*4 (trailer axle group spacing, where applicable), and *d*5 (trailer wheelbase) [18]. The distribution of these longitudinal spacings is presented in Fig 3(b).

**Fig 4:**
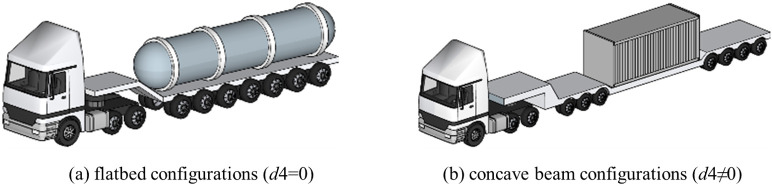
Typical layout of restricted overload vehicles.

Generally, the axles corresponding to *d*1 and *d*2 primarily support the self-weight of the tractor, employ a single-line arrangement, and have a single-axle load capacity of 10 t. Trailers are mainly responsible for carrying cargo weight: a single-line configuration with a 10 t load limit is used for light cargo, while a double-line arrangement is employed for heavy cargo, allowing the load capacity to be increased to 18 t. Currently, mainstream ROVs on the market are universally equipped with hydraulic balancing devices, which can dynamically adjust the load distribution across all trailer wheels to achieve uniform load bearing. Furthermore, depending on the presence of *d*4, ROVs can be further classified into flatbed configurations (*d*4 = 0) and concave beam configurations (*d*4 ≠ 0), as shown in Fig 4. Flatbed ROVs are typically used to transport short cargo (e.g., precast beams, hoisting machinery), with lengths mostly ranging from 10–20 m; concave beam ROVs are suitable for long cargo (e.g., wind turbine blades, train carriages), with lengths generally exceeding 30 m. The mass of such cargo is often not excessive, so it is unnecessary to arrange wheels along the entire length of the trailer. Carrier units can reasonably determine the number of axles in compliance with load limit standards and vehicle power performance. According to market research and permit data statistics, flatbed ROVs account for over 90% of the total, whereas concave beam ROVs represent less than 10%. Among flatbed configurations, more than 90% of trailers adopt a double-line arrangement, with a load capacity of 18 t.

Nevertheless, it should be acknowledged that ROV parameters can vary across regions. For instance, vehicles transporting wind turbine blades in western provinces may exhibit considerably greater lengths and a larger number of axles, which may not be fully captured by the present dataset. Therefore, when applying the model to other regions, it is recommended to fine-tune or retrain it using local or more recent permit data. To facilitate such updates, the complete model training code has been made available in the data repository, allowing other researchers to adapt the model with their own data.

## 4. Setting of mixed traffic ROVs and regular vehicles

To systematically evaluate the impact of combined ROV and regular vehicle loads on bridges under open traffic, it is essential to establish well-designed, representative analysis scenarios. This chapter elaborates on the load scenario construction approach employed in this research from three aspects: the simulation approach for regular vehicle loads, the selection of representative bridge samples, and the specific arrangement principles for mixed traffic loads on the bridge deck.

### 4.1 Regular vehicle loads

In bridge safety assessment, there are two primary approaches for simulating regular vehicle loads: one is to employ a probability statistics-based random traffic flow model to replicate the actual traffic condition [19,20]; the other is to utilize the design vehicle loads specified in technical specifications, which offers advantages such as standardized criteria, ease of engineering application, and explicit code comparability of results. To guarantee the broad applicability of the analysis findings and uphold the safety-prioritized design and assessment principle, this study utilizes standardized design vehicle loads to simulate regular vehicle loads in mixed traffic. Specifically, it selects load classes widely adopted in the design of expressways and key trunk line bridges in China, including Automobile-Super 20 and Highway-Class I.

(1) **Automobile-Super 20**

**Fig 5:**
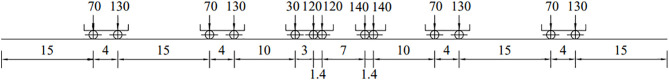
Automobile-Super 20 (axle load unit: kN, distance unit: m).

In the *General Specifications for Design of Highway Bridges and Culverts* (JTJ 021–89, implemented in 1989) [21], design vehicles are defined as fleet loads, which are categorized into four levels based on load intensity. Each load level is simulated using fleet loads of two-axle, three-axle, and five-axle vehicles, and the modeling approach is intuitive and straightforward. For important highways (e.g., expressways and national-provincial trunk lines) characterized by heavy traffic volumes, the design vehicle load typically employs the Automobile-Super 20 class. This fleet load class comprises a five-axle heavy vehicle with a total weight of 55 t; therefore, the Automobile-Super 20 fleet load is selected to simulate the regular vehicle load in mixed traffic. The schematic diagram for the calculation of the Automobile-Super 20 fleet load is presented in Fig 5.

(2) **Highway-Class I**

**Fig 6:**
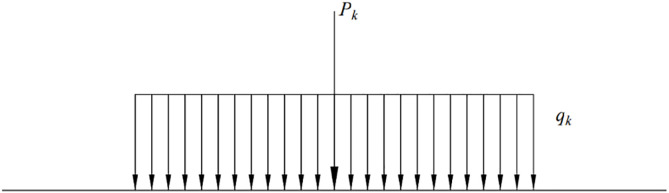
Lane load.

With socioeconomic development and the continuous growth of highway traffic volumes, the *General Specifications for Design of Highway Bridges and Culverts* (JTG D60-2004, implemented in 2004) [22] and the *General Specifications for Design of Highway Bridges and Culverts* (JTG D60-2015, implemented in 2015) [23] categorize vehicle load levels into Highway-Class I and Highway-Class II, and divide load types into lane loads and vehicle loads. Lane loads are employed for the global inspection of bridges (as illustrated in Fig 6), whereas vehicle loads are utilized for local inspections. Both expressways and first-class highways adopt Highway-Class I as their design vehicle load level. In subsequent calculations, the Highway-Class I lane loads specified in JTG D60-2004 and JTG D60-2015 are abbreviated as 04 Highway-Class I and 15 Highway-Class I, respectively.

The uniform lane load intensity *q*_*k*_ for Highway-Class I specified in the JTG D60-2004 and the JTG D60-2015 is 10.5 kN/m, while the standard values of the concentrated load *P*_*k*_ are presented in Table 1.

**Table 1:** Concentrated load *P*_*k*_ values for lane loads in JTG D60-2004 and JTG D60-2015.

Specification	*L*_0_ ≤ 5	5 < *L*_0_ < 50	*L*_0_ ≥ 50
JTG D60-2004	180	4(L_0_ + 40)	360
JTG D60-2015	270	2(L_0_ + 130)	360

### 4.2 Selection of typical bridges

To ensure the research findings cover common small- and medium-span bridges in China’s highway network, this study selected 17 representative precast concrete beam bridges as analytical objects in compliance with the *General Drawings of Highway Bridge Superstructures* issued by the Ministry of Transport of the People’s Republic of China. The selected bridge types include: Reinforced Concrete Simply Supported Hollow Slab Bridge (RCS), Prestressed Concrete Simply Supported Hollow Slab Bridge (PCS), Prestressed Concrete Simply Supported T-Beam Bridge (PCT), and Prestressed Concrete Continuous Box Girder Bridge (PCB). The calculation span ranges from 6 m to 40 m (for continuous beam bridges, the span configuration ranges from 4 × 20 m to 4 × 40 m), essentially covering the commonly adopted spans for this category of bridges [18]. The geometric parameters of individual girders for representative bridges are presented in Table 2.

**Table 2:** Geometric parameters of representative bridges.

Cross-section	Span (*m*)	Depth of beam (*m*)	Cross-Sectional Area (*m*^2^)	Longitudinal Moment of Inertia (*m*^4^)	Transverse Moment of Inertia (*m*^4^)	Torsional Moment of Inertia(*m*^4^)
RCS	6	0.32	0.39	0.015	0.006	0.028
	8	0.42	0.40	0.024	0.011	0.030
	10	0.50	0.44	0.031	0.016	0.032
PCS	10	0.60	0.55	0.061	0.031	0.080
	13	0.70	0.59	0.087	0.044	0.086
	16	0.80	0.62	0.109	0.060	0.093
	20	0.95	0.67	0.144	0.090	0.105
PCT	20	1.58	0.96	0.023	0.268	0.232
	25	1.78	1.01	0.024	0.269	0.334
	30	2.08	1.08	0.025	0.270	0.515
	35	2.38	1.17	0.027	0.271	0.798
	40	2.58	1.28	0.036	0.275	1.098
PCB	4 × 20	1.20	1.27	0.337	0.676	0.220
	4 × 25	1.40	1.34	0.430	0.704	0.323
	4 × 30	1.60	1.42	0.524	0.732	0.450
	4 × 35	1.80	1.49	0.659	0.779	0.604
	4 × 40	2.00	1.63	0.848	0.853	0.809

**Fig 7:**
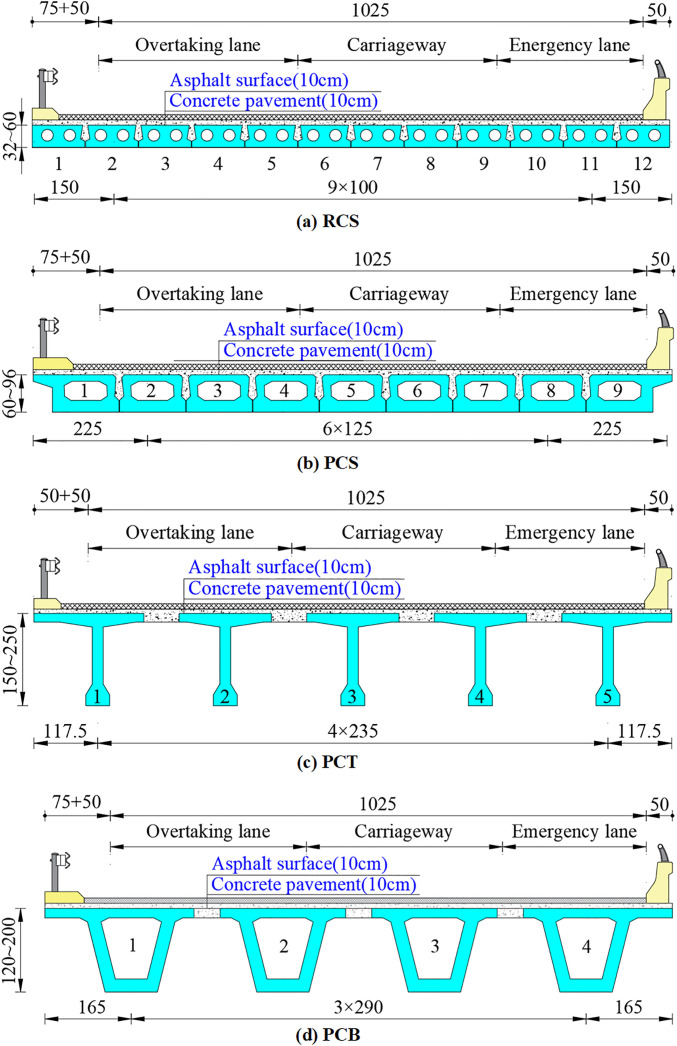
Typical midspan cross-sections of various bridge types (unit: cm).

Hollow slab bridges feature a constant cross-section longitudinally, while simply supported T-beam bridges and continuous box girder bridges adopt a variable cross-section along the longitudinal direction, with the web thickness of the main girders thickened at the supports. Taking bridge types with a deck width corresponding to three design lanes as an example, the mid-span cross-sections of each bridge type are illustrated in Fig 7.

### 4.3 Principles of transverse arrangement of mixed loads

The transverse arrangement of loads on the bridge deck directly governs the force distribution among the main girders. Based on observations of the driving behaviors of ROVs on actual expressways, they typically travel along the outermost traffic lane (slow lane). Accordingly, this study formulates the following loading principles: (1) ROVs default to traveling along the centerline of the outermost unidirectional traffic lane; (2) On each traffic lane inside this ROV lane, the corresponding lane load is applied in accordance with the selected design load classes (Automobile-Super 20, 04 Highway-Class I, or 15 Highway-Class I), and the transverse reduction coefficient for multi-lane loads is considered in compliance with relevant specifications; (3) No load is applied to the emergency lane under normal traffic conditions. When an ROV actually needs to occupy two lanes due to excessively large cargo width, the number of lanes available for regular vehicles inside the ROV’s occupied lanes is reduced accordingly. This arrangement mode simulates a common traffic scenario that may exert relatively adverse effects on the main girders beneath the ROV, serving to systematically analyze the spatial coupling effect induced by the combined loads of ROVs and parallel regular vehicles. Nevertheless, actual mixed traffic may involve different lane positions, densities, or control measures. The above assumption (ROV on outermost lane and design loads on all other lanes) is a conservative simplification for permit assessment, targeting the most unfavorable case. If the ROV travels on inner lanes or traffic is sparse, load effects would be smaller; the framework remains applicable by redefining lane assignments.

In addition, this study adopts code-specified design loads to represent conventional vehicles, thereby ensuring the comparability of evaluation results and a high level of safety redundancy. However, this approach may overestimate the actual traffic load effects. Since the evaluation method proposed in this paper is primarily intended for the rapid preliminary screening of mixed traffic consisting of ROVs and conventional vehicles, a more realistic approach to constructing mixed vehicle load models can be employed for ROVs that fail the preliminary screening or carry exceptionally important cargo. The current mainstream approach for refined traffic load simulation utilizes measured traffic flow data collected by weigh-in-motion (WIM) systems in conjunction with microscopic traffic simulation models [20,24,25] to generate actual vehicle platoon load sequences. The corresponding mixed-traffic effects can then be calculated using the influence surface loading method proposed in this paper. Due to the research objectives and length limitations, this paper considers only the conservative simulation method based on code-specified design loads. Future work will further integrate the refined microscopic simulation framework with the machine learning prediction model to enhance the accuracy and comprehensiveness of the evaluation framework.

## 5. Analytical method for small- and medium-span bridges under mixed traffic of ROVs and regular vehicles

### 5.1 Establishment of bridge finite element model

**Fig 8:**
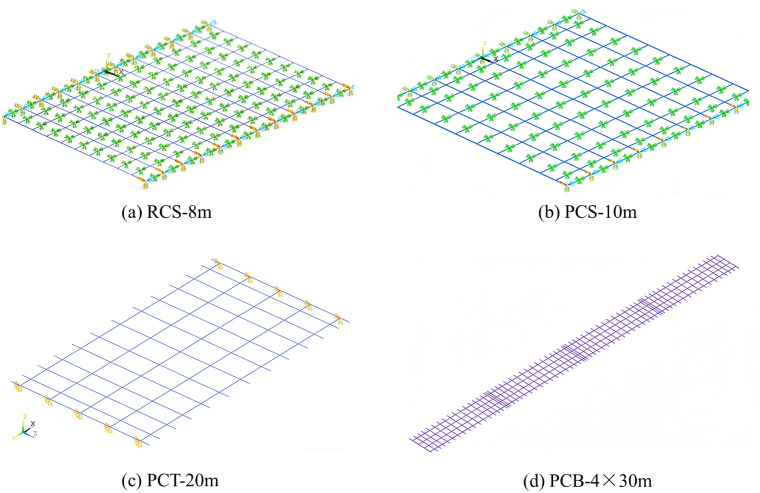
Finite element models of small- and medium-span beam bridges.

ANSYS finite element analysis software is employed to construct the grillage models of bridge structures, in which both main girders and cross girders are modeled using BEAM-4. Hinged joints in hollow slab bridges were simulated by coupling nodal translational displacements while releasing rotational degrees of freedom. Cast-in-place joints in T-beam and box girder bridges were modeled as integral parts of the girder flanges. Considering the effect of bridge deck pavement on the moment of inertia of main girders in small- and medium-span beam bridges, the concrete pavement layer is incorporated as part of the cross-sectional top slab, while the effect of the asphalt pavement layer is neglected. The accuracy of the bridge finite element model constructed using the aforementioned method has been validated in prior studies [26,27]. Taking the 8 m Reinforced Concrete Simply Supported Hollow Slab Bridge (RCS), 10 m Prestressed Concrete Simply Supported Hollow Slab Bridge (PCS), 20 m Prestressed Concrete Simply Supported T-Beam Bridge (PCT), and 4 × 30 m Prestressed Concrete Continuous Box Girder Bridge (PCB) as representative cases, the grillage models for bridges with a deck width corresponding to three design lanes are presented in Fig 8.

### 5.2 Influence surface-based moving load application method

To further explore the characteristics of bridge load responses under mixed traffic conditions of ROVs and regular vehicles, this study necessitates the development of refined grillage models for load response analysis. However, the traditional moving load application method exhibits notable limitations: it typically entails the independent development of complex custom loading subroutines tailored to specific bridges and load scenarios, leading to low modeling efficiency; more critically, this method fails to efficiently implement synchronous dynamic loading analysis of massive moving loads (particularly complex mixed vehicle fleets) across multiple bridge structures, resulting in an excessively time-consuming calculation process and substantial computational resource consumption.

To overcome these bottlenecks, this study employs an efficient moving load analysis method based on influence surfaces. Its core concept is implemented through the following steps: First, by calculating the refined grillage model detailed in Section 4.1, the load influence surfaces of key control sections of the target bridge (e.g., the mid-span maximum positive bending moment section, support maximum negative bending moment section, and support maximum shear force section) are extracted. These influence surfaces precisely characterize the response values induced at the control sections when a unit load is applied to different positions on the bridge deck. Subsequently, in the MATLAB environment, utilizing the extracted influence surface data, the dynamic response histories (e.g., bending moment and shear force time histories) induced at these key control sections by any moving load sequence (including large-scale mixed formations of ROVs and regular vehicles) traversing the bridge are directly computed through efficient matrix operations. Leveraging the pre-calculated influence surfaces, batch analysis of load responses for various vehicle fleet combinations and different bridge structures can be achieved, which avoids the cumbersome iterative loading processes and complex subroutine invocations inherent to the traditional method, while substantially enhancing computational efficiency.

Based on the finite element model established in Section 5.1, the influence surfaces for key responses at key control sections are extracted, and the extraction procedure is as follows: First, the key control sections of each main girder are precisely defined. For simply supported beams, the mid-span positive bending moment section is situated at the mid-span of each main girder, while the support shear force section is located at the beam end on one side of the support, within a distance not exceeding 0.5 times the beam height from the support centerline. For continuous beams, the positive bending moment section is designated at 0.4*L* from the intermediate support of the minor side span (where *L* denotes the span length of the minor side span), and the key negative bending moment control section corresponds to the support section of the minor side span. The unit load is normalized as a concentrated force (P = 1), adopting a longitudinal step size of Δ*x* = 0.25 m (with the longitudinal step size refined to 0.1 m in the negative moment regions of continuous beams) and a transverse step size equivalent to the spacing between adjacent main girders; the load movement range covers the entire bridge deck. During the full bridge deck scanning process, a unit load is applied individually at each discrete point, and the section response values of all main girders are batch-extracted subsequent to the static analysis of each load case.

**Fig 9:**
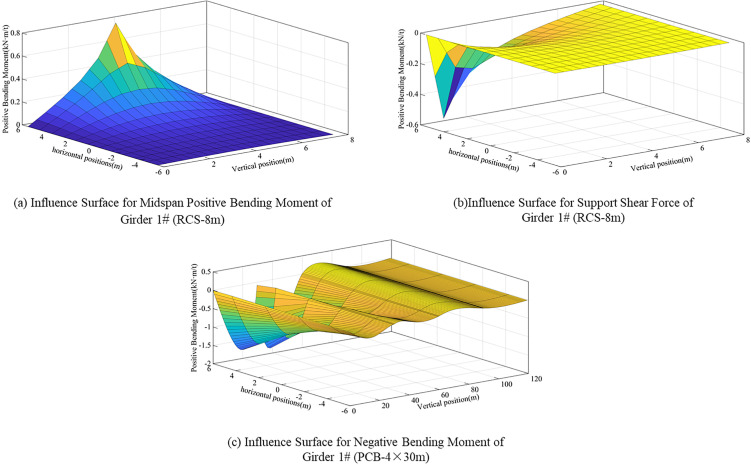
Influence surfaces for internal force responses at key sections of typical bridges.

Taking Girder 1# of the RCS-8m as an example, the influence surfaces for mid-span positive bending moment and support shear force of the girder are computed. Additionally, taking Girder 1# of the PCB-4 × 30m as a representative case, the influence surface for negative bending moment of the girder is calculated. The calculated influence surfaces are presented in Fig 9.

## 6. Analysis of load effect distribution characteristics of typical small- and medium-span bridges under mixed traffic of ROVs and regular vehicles

### 6.1 Load information of five typical ROVs

Load data of over 2,000 ROVs were collected in Chapter 2. To facilitate the analysis of the distribution characteristics of load effects induced by ROVs under mixed traffic conditions of ROVs and regular vehicles on typical small- and medium-span bridges, five typical ROVs are selected in this chapter based on axle load distribution and total vehicle-and-cargo weight. The load parameters are presented in Table 3.

**Table 3:** Load parameters of five typical ROVs.

Vehicle Number	Total Weight (t)	Axle Load Distribution (t)	Wheelbase Distribution(m)
1	119.00	10.00 + 10.00 + 10.00 + 5 × 17.80	3.50 + 1.50 + 8.00 + 4 × 1.25
2	150.00	10.00 + 10.00 + 10.00 + 7 × 17.14	3.00 + 1.20 + 5.50 + 6 × 1.55
3	169.00	10.00 + 10.00 + 10.00 + 9 × 15.44	3.00 + 1.50 + 4.00 + 8 × 1.55
4	180.00	8.00 + 8.00 + 8.00 + 10 × 15.60	3.50 + 1.40 + 3.10 + 9 × 1.55
5	200.00	8.00 + 8.00 + 8.00 + 16 × 11.00	3.90 + 1.35 + 3.72 + 10 × 1.51

### 6.2 Analysis of load effects induced by five typical ROVs crossing bridges alone

Based on the influence surface-based moving load application method established in Section 4.2, load response analyses were conducted for the aforementioned five typical ROVs traversing the representative small- and medium-span bridges listed in Table 2 (featuring three design lanes), where the ROVs were assumed to travel in the slow lane. The calculated results of positive bending moment, shear force, and negative bending moment at the key control sections of each bridge type are presented in –. The key findings are: (1) Bridge load responses generally increase with span length; (2) For hollow slab bridges, the load effects induced by low-weight ROVs are relatively more significant for short-span configurations. This phenomenon is associated with the axle load distribution of the 119-ton ROV, although its total weight is relatively small, the trailer axle load reaches 18 tons per axle. Additionally, short-span bridges have a relatively small number of effectively loaded axles, thereby leading to more pronounced load effects. Consequently, greater attention should be paid to axle load distribution rather than merely total weight during practical inspection and calculation work; (3) For short-span bridges, the dispersion of load effects induced by different ROVs is relatively low. With the increase in span length and bridge length, the number of axles contributing to the load increases, and the dominance of load effects induced by large-tonnage ROVs gradually becomes evident; (4) From a numerical standpoint, the load effects induced by the selected typical ROVs are relatively close to those induced by the design vehicle load, posing substantial potential safety risks to the bridges under traversal.

**Fig 10:**
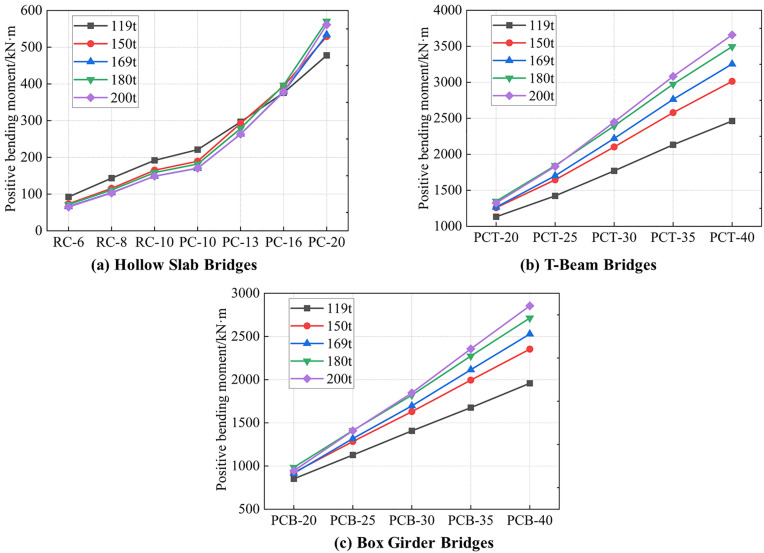
Variation curves of positive bending moment by five typical ROVs.

**Fig 11:**
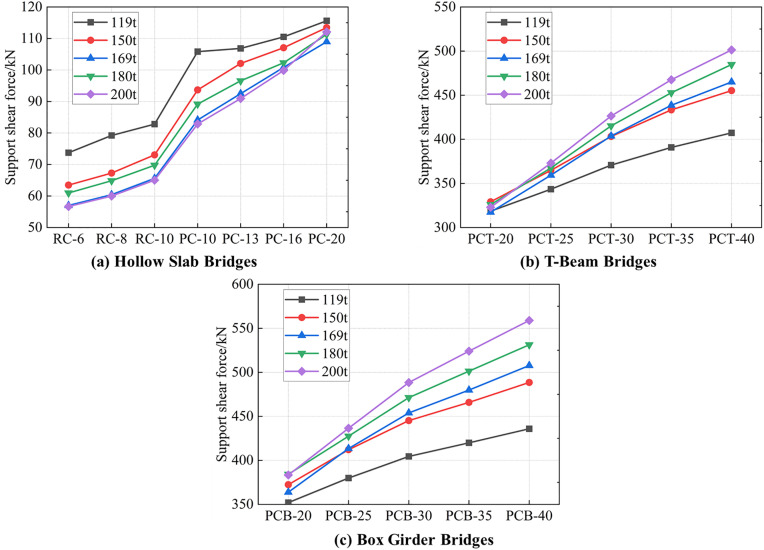
Variation curves of support shear force by five typical ROVs.

**Fig 12:**
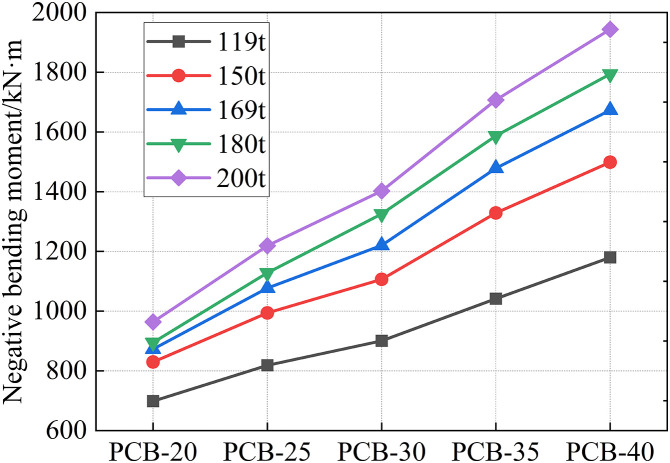
Variation curves of negative bending moment by five typical ROVs.

### 6.3 Analysis of load effects in mixed traffic of ROVs and regular vehicles

Based on the load effects of ROVs traversing solo calculated previously, the design vehicle load effects are superimposed. The superposition calculation adopts the same methodology as that for ROVs traversing alone: design vehicle loads are applied to all lanes except the one occupied by the ROV (including the emergency lane), with the corresponding lane coefficients and design vehicle load impact coefficients considered. To analyze the distribution characteristics of bridge load responses under mixed traffic loads with ROVs, the load effect amplification factor *ξ* for bridges under mixed traffic is defined as the ratio of the extreme value of bridge load effects under mixed traffic conditions to that under solo ROV traversal conditions:


$$\begin{array}{c} \zeta =\frac{S_{\text{mix}}}{S_{OTV}} \end{array}$$ (1)

In the formula, *S*_mix_ represents the load effect values of highway bridges under the mixed passage of oversized vehicles and regular vehicles, specifically including positive bending moment, negative bending moment, and support shear force. *S*_ROV_ denotes the load effect values when oversized vehicles pass alone. Both calculations account for the impact effects induced by oversized vehicles and regular vehicles. The impact factor for regular vehicles is selected based on current bridge design specifications, while the impact factor for oversized vehicles is determined based on the research findings of the research group [18].

Taking the Automobile-Super 20 class design vehicle load as an example, the analysis results of the load effect amplification factor *ξ* for five typical ROVs traversing representative bridges under mixed traffic conditions are presented in –. Analysis results indicate that mixed traffic load effects are primarily influenced by factors including bridge type, span length, and axle load distribution characteristics. The specific influence mechanisms are as follows:

**Fig 13:**
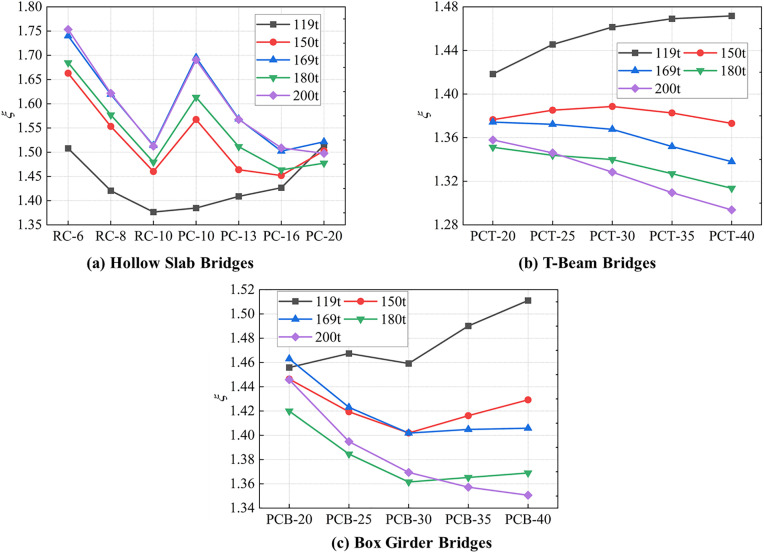
Positive bending moment load effect amplification factors for typical bridges under mixed traffic of five typical ROVs.

**Fig 14:**
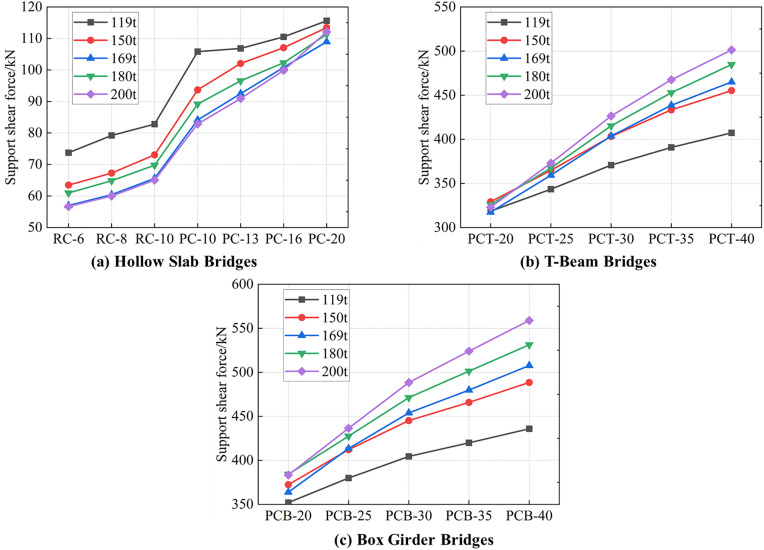
Support shear force load effect amplification factors for typical bridges under mixed traffic of five typical ROVs.

**Fig 15:**
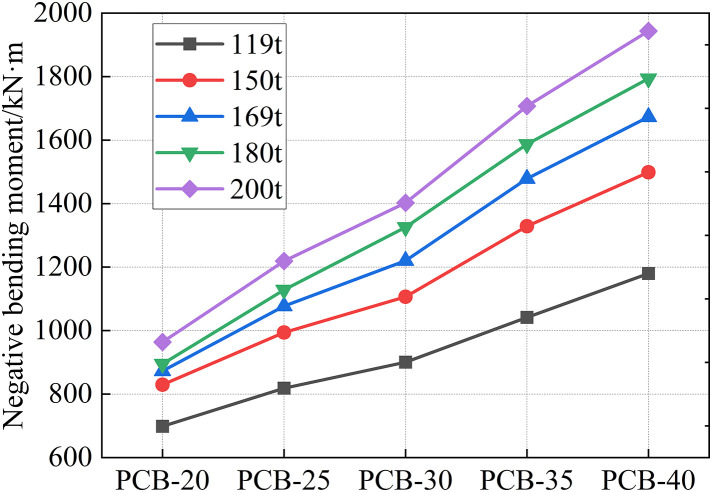
Negative bending moment load effect amplification factors for typical bridges under mixed traffic of five typical ROVs.

(1) Influence of bridge type: The load effect amplification factor *ξ* of hollow slab bridges is significantly higher than that of T-beam bridges and box girder bridges. This discrepancy is primarily associated with the effective span characteristics of the respective bridge types.
(2) Combined influence of span length and ROV type: For short-span bridges, low-weight ROVs (characterized by small total vehicle-and-cargo weight, few axle groups, and short overall length) result in smaller *ξ* values. This is because such vehicles can be fully accommodated on the bridge deck for short spans, whose load effects account for a relatively high proportion of the total mixed traffic load effects. However, as span length increases, the load effects of low-weight ROVs increase relatively slowly, leading to a gradual decrease in their contribution to the mixed effects; correspondingly, the *ξ* values induced by these ROVs increase instead. For high-weight ROVs (featuring large total vehicle-and-cargo weight, numerous axle groups, and long overall length), the opposite trend is observed. With increasing span length, more axles of these vehicles can be effectively loaded on the bridge deck, their load effects grow rapidly, and their proportion in the mixed traffic load effects increases accordingly. Thus, for long-span bridges, high-weight ROVs correspond to smaller *ξ* values.
(3) Non-dominant role of total vehicle-and-cargo weight: For bridges with most span lengths, the greater the total weight, the smaller the load effect amplification factor. Nevertheless, not all *ξ* values decrease with increasing total weight; the variation trend is more critically determined by the specific distribution characteristics of the vehicle’s axle loads.

## 7. Prediction method for the mixed traffic load effect amplification factor based on machine learning

### 7.1 Dataset for the load effect amplification factor *ξ* under mixed traffic loads with ROVs

The load effect amplification factors of five high-frequency ROVs under mixed traffic with regular vehicles were computed in Chapter 5. In this chapter, a customized batch loading program for ROVs and regular vehicles was developed using MATLAB software to further calculate and analyze the mixed traffic load effect amplification factors of over 2,000 ROVs traversing 17 representative small- and medium-span bridges (featuring three design lanes). The regular vehicle loads adopted include the Automobile-Super 20 class, 04 Highway-Class I, and 15 Highway-Class I. The distributions of the load effect amplification factors under mixed traffic of ROVs with these three types of regular vehicle loads are presented in –. Analysis indicates that the *ξ* distributions under all working conditions exhibit an obvious approximate normal distribution. When ROVs operate under mixed traffic with the 15 Highway-Class I load, the mean and maximum values of the load effect amplification factor are the highest in most cases, among which the maximum shear force *ξ* reaches 4.09, indicating that the extreme load effects induced by this load combination are the most pronounced. Under all working conditions, the mean *ξ* value for positive bending moment is the lowest, confirming that it exhibits the least load effect amplification. Under the two types of Highway-Class I loads, the mean *ξ* values for negative bending moment and shear force are similar; whereas under the Automobile-Super 20 class load, the mean *ξ* value for negative bending moment (1.81) is the highest, while that for shear force (1.44) is the lowest, indicating that the negative bending moment response is more sensitive to this specific load combination.

**Fig 16:**
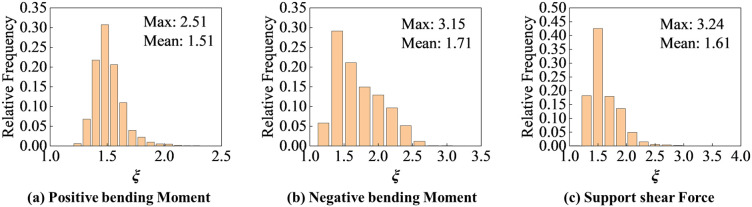
Load effect amplification factor *ξ* under mixed traffic of ROVs and 04 Highway-Class I load.

**Fig 17:**
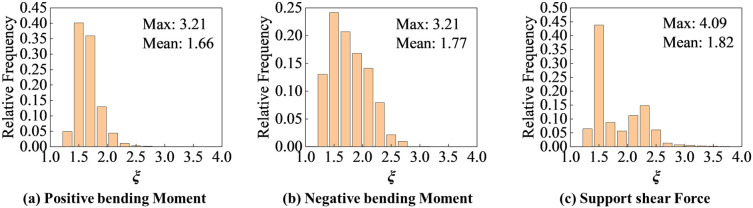
Load effect amplification factor *ξ* under mixed traffic of ROVs and 15 Highway-Class I load.

**Fig 18:**
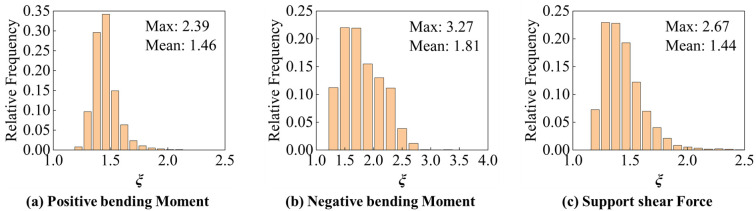
Load effect amplification factor *ξ* under mixed traffic of ROVs and Automobile-Super 20 class load.

Based on the aforementioned calculation results, this study establishes a dataset where the load effect amplification factor *ξ* serves as the target output, and the ROV load distribution parameters and key bridge parameters are used as input features. Specifically, the ROV load distribution parameters include: average tractor axle weight, average trailer axle weight, number of trailer axles (with a per-axle load limit of 18 t), five wheelbase parameters (*d*1–*d*5), and total wheelbase, resulting in a total of 9-dimensional features. The key bridge parameters include: span length, total bridge length, longitudinal moment of inertia of the main girder, transverse moment of inertia of the main girder, torsional moment of inertia of the main girder, and cross-sectional area of the main girder, resulting in a total of 6-dimensional features. The main statistical characteristics of the ROV and bridge-related feature parameters utilized in this study are presented in Table 4. Except for a difference in the number of bridges considered for negative bending moment responses (only continuous box girder bridges produce negative bending moment responses), the distributions of feature parameters under other mixed traffic load conditions and internal force response types are consistent.

**Table 4:** Key influencing factors of load effect amplification factor *ξ* under mixed traffic loads with ROVs.

Feature serial number	Feature name	Unit	Minimum value	Maximum value	Average value
1	Tractor Axle Weight	ton	8.00	10.00	9.99
2	Trailer Axle Weight	ton	11.00	18.00	17.98
3	Number of Trailer Axles	/	4.00	18.00	6.71
4	Wheelbase 1	m	1.00	4.2.	3.25
5	Wheelbase 2	m	0.40	1.86	1.38
6	Wheelbase 3	m	1.50	31.00	9.02
7	Wheelbase 4	m	0.00	27.00	1.86
8	Wheelbase 5	m	0.82	1.95	1.32
9	Total Wheelbase	m	10.00	61.2	23.09
10	Bridge Span	m	6.00	40.00	22.53
11	Total Bridge Length	m	6.00	160.00	49.00
12	Longitudinal Moment of Inertia	m^4^	0.015	0.853	0.328
13	Transverse Moment of Inertia	m^4^	0.006	0.848	0.188
14	Torsional Moment of Inertia	m^4^	0.028	1.098	0.343
15	Cross-Sectional Area	m^2^	0.390	1.630	0.959

### 7.2 Modeling process for predicting the mixed traffic load effect amplification factor with ROVs

Using the input features defined in Section 6.1, a dataset for the load effect amplification factor was constructed by matching ROVs and bridge parameters with the corresponding simulation results. According to different regular vehicle load levels in mixed traffic and internal force response types, a total of 9 sub-datasets are constructed in this study. The overall modeling process is as follows:

(1) Dataset splitting and preprocessing: A consistent splitting strategy is employed, where samples are divided into a training set and a test set at a 7:3 ratio with a fixed random seed to ensure benchmark consistency across all comparative experiments. Meanwhile, feature data are standardized to eliminate dimensionality-induced biases.
(2) Hyperparameter optimization: Seven representative machine learning algorithms are selected for modeling and analysis, including Random Forest (RF), Gradient Boosting Tree (GBT), AdaBoost (ADB), Multi-Layer Perceptron (MLP), K-Nearest Neighbors (KNN), XGBoost (XGB), and LightGBM (LGB) [28]. To reduce computational overhead, this study adopts a hyperparameter tuning approach combining the hold-out method and cross-validation, strictly separating the training and test sets. Hyperparameter tuning is implemented via cross-validation on the training set, while performance evaluation is conducted on the test set.

Specifically, 5-fold cross-validation is performed on the training set, and the Bayesian optimization framework [29,30] (with 100 iterations) is utilized to conduct parameter search, aiming to maximize the average negative mean squared error from the 5-fold cross-validation. Finally, the optimal hyperparameters derived from the search are used for performance evaluation on a fully independent test set. This ensures the test set does not participate in any form of model training or hyperparameter selection, thereby yielding an unbiased performance estimation.

(3) Model training and evaluation: The models are trained on the training set using the optimal hyperparameters, and a multi-dimensional evaluation framework is established. The coefficient of determination (*R*²), root mean square error (*RMSE*), and mean absolute error (*MAE*) are selected as evaluation metrics [29,31], and their specific calculation formulas are presented as follows:

$$\begin{array}{c} R^{\text{2}}=1-\sum_{i=1}^{n}(Y_{i}-{\hat{Y}}_{i}{)}^{\text{2}}/\sum_{i=1}^{n}(Y_{i}-\overset{―}{Y}{)}^{\text{2}} \end{array}$$ (2)

$$\begin{array}{c} RMSE=\sqrt{\frac{\text{1}}{n}\sum_{i=1}^{n}(Y_{i}-{\hat{Y}}_{i}{)}^{\text{2}}} \end{array}$$ (3)

$$\begin{array}{c} MAE=\frac{\text{1}}{n}\sum_{i=1}^{n}|Y_{i}-{\hat{Y}}_{i}| \end{array}$$ (4)

In the formula, *Y*_*i*_ represents the *i*_th_ actual observed value, $\hat{Y}$ is the corresponding predicted value, *n* is the total number of observations, $\overset{―}{Y}$ is the average of the actual observed values.

### 7.3 Result analysis

**Fig 19:**
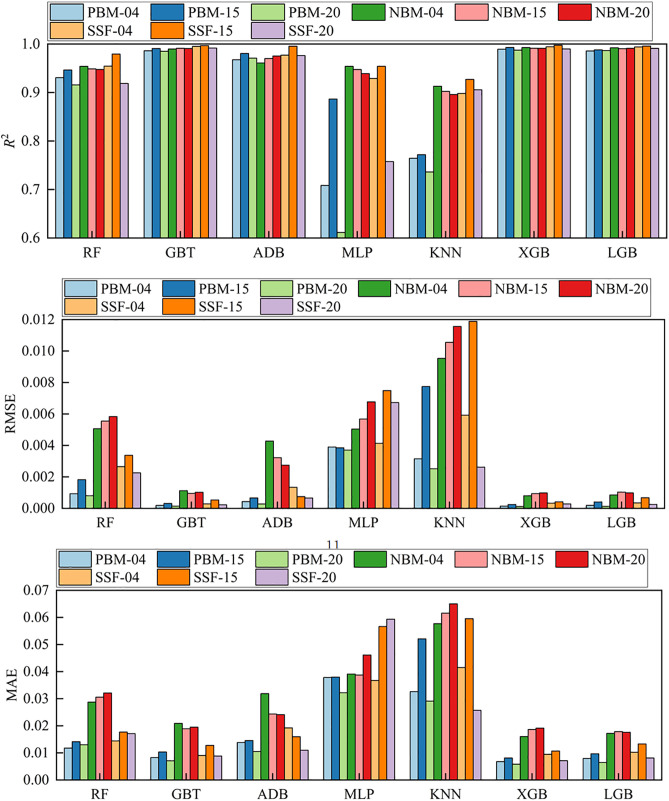
Performance indicators of machine learning model under different mixed traffic load levels.

Based on the modeling process outlined in Section 7.2, consistent dataset splitting and model training are conducted on the 9 sub-datasets. The prediction results of the load effect amplification factor *ξ* under different mixed traffic load conditions and internal force response types are obtained through systematic calculations. The prediction performance metrics of each machine learning model on the test set are presented in Fig 19. For brevity, Positive Bending Moment is abbreviated as PBM, Negative Bending Moment as NBM, and Support Shear Force as SSF; 04 Highway-Class I load is abbreviated as 04, 15 Highway-Class I load as 15, and Automobile-Super 20 class load as 20. The 9 sub-datasets are numbered corresponding to the combinations of internal force response types and mixed traffic load levels. The following conclusions can be drawn from Fig 19:

(1) In most sub-datasets, RF, GBT, XGB, and LGB demonstrate high prediction accuracy and stability. Their *R*² are generally greater than 0.9, while the *RMSE* and *MAE* remain at low levels. This reflects that tree-based ensemble learning methods possess robust capabilities in capturing the nonlinear correlations between load parameters and amplification factors. In contrast, MLP and KNN exhibit significant performance fluctuations across certain datasets. Particularly in the NBM and SSF-related sub-datasets, their prediction errors increase markedly, indicating poor adaptability to the data distribution characteristics and feature attribute patterns of these scenarios.
(2) All models achieve generally satisfactory prediction performance on the PBM and SSF sub-datasets, with most *R*² values exceeding 0.95. This indicates a relatively clear mapping relationship between these internal force responses and load parameters, which is conducive to model training. In contrast, the prediction performance of all models degrades to varying degrees on the NBM sub-datasets. This phenomenon may be attributed to the small sample size and high data variability of negative bending moment responses (only continuous box girder bridges exhibit such responses). Consequently, more sophisticated feature engineering or targeted model structure optimization is required to achieve accurate prediction of NBM-related amplification factors.

Based on the test set prediction results, Table 5 presents the optimal machine learning models and their corresponding performance metrics for each combination of mixed traffic loads and internal force responses, while Fig 19 illustrates the scatter distribution of predicted values versus actual values on the test set. Table 5 reveals that except for the two datasets corresponding to the SSF-04 and SSF-20 combinations, the XGB model delivers the optimal prediction performance across the remaining seven scenarios. The predicted *R*² for all datasets exceeds 0.98, and the *MAE* is controlled within 0.02. This indicates that the XGB’s prediction accuracy fully meets the engineering calculation requirements for the load effect amplification factor *ξ* in the mixed traffic of ROVs and regular vehicles in practical engineering applications.

**Table 5:** Optimal models and performance indicators under different mixed traffic load levels.

Dataset	Optimal Model	Train Set			Test Set		
		*R* ^2^	*RMSE*	*MAE*	*R* ^2^	*RMSE*	*MAE*
PBM-04	XGBoost	0.9988	1.68E-05	0.0027	0.9894	1.41E-04	0.0068
PBM-15	XGBoost	0.9996	1.48E-05	0.0025	0.9928	2.44E-04	0.0081
PBM-20	XGBoost	0.9997	2.93E-06	0.0012	0.9874	1.20E-04	0.0058
NBM-04	XGBoost	0.9997	3.70E-05	0.0042	0.9927	8.02E-04	0.0160
NBM-15	XGBoost	0.9991	1.03E-04	0.0069	0.9913	9.38E-04	0.0186
NBM-20	XGBoost	0.9984	1.86E-04	0.0089	0.9912	9.80E-04	0.0191
SSF-04	GradientBoost	0.9996	2.70E-05	0.0032	0.9951	2.86E-04	0.0090
SSF-15	XGBoost	0.9998	4.25E-05	0.0042	0.9974	4.16E-04	0.0107
SSF-20	GradientBoost	0.9996	1.22E-05	0.0024	0.9918	2.29E-04	0.0088

**Fig 20:**
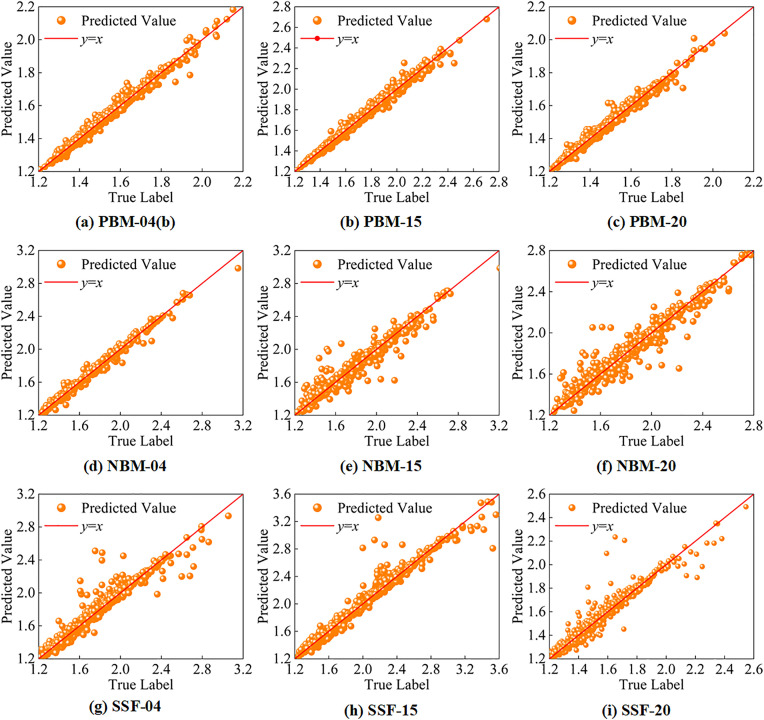
Predicted values versus actual values for each sub-dataset under the respective optimal models.

Fig 20’s scatter plots reveal that the predicted values of all sub-datasets are closely clustered around the reference line (*y* = *x*), further validating the overall reliability of the optimal models. Notably, the predicted results for positive bending moment responses are the most tightly clustered, while the negative bending moment responses exhibit a moderately higher degree of dispersion. Additionally, a small subset of prediction points for support shear force data exhibits substantial deviations. This observation suggests inherent differences in prediction stability across different internal force response types, with NBM prediction posing a relatively greater challenge compared to PBM and SSF.

### 7.4 Explanation of the optimal model

To thoroughly elucidate the decision-making mechanism of the prediction model for the amplification factor of bridge load effects under mixed traffic, the SHAP (Shapley Additive Explanations) method is introduced in this study to perform feature interpretation analysis on the optimal prediction model (XGBoost) identified in Section 6.3. Grounded in cooperative game theory, the SHAP method achieves a fair allocation of the model’s prediction outcomes by calculating the average marginal contribution of each feature across all possible combinations of feature subsets. The output, represented as SHAP values for each feature, quantifies both the direction and magnitude of each feature’s contribution to the prediction, thereby providing a theoretical basis for understanding the internal working mechanism of the model.

**Fig 21:**
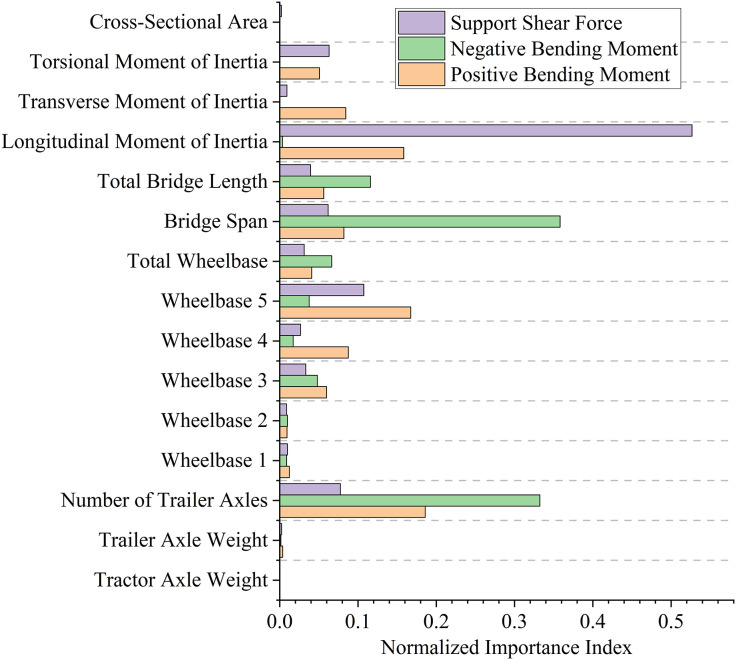
Normalized importance indexes of each input feature parameter.

Based on the complete dataset constructed in Section 6.1, SHAP analyses were conducted for nine combination scenarios, encompassing three response types (positive bending moment, negative bending moment, and shear force) and three load classes (04 Highway-Class I, 15 Highway-Class I, and Motorway-Overload 20). The mean absolute SHAP value of each feature is adopted as the metric for feature importance, where a larger value indicates a greater contribution of the feature to the model output. To facilitate comparison of the contribution differences of characteristic parameters under various load response types, the SHAP analysis results for the three response types were aggregated. The feature importance metrics for each response type were then calculated and normalized based on their proportional contributions. The resulting normalized feature importance is illustrated in Fig 21.

Based on the SHAP analysis results, the following insights are obtained: (1) The number of trailer axles plays a dominant role in the responses of positive bending moment, negative bending moment, and shear force. This is because the axle count directly dictates the total load magnitude and its longitudinal distribution length along the bridge span. A higher number of axles increases the load scale and intensifies the superposition effect, thereby amplifying the peak responses of the mid-span moment, support negative moment, and support shear force. (2) The top three features influencing the positive bending moment and shear force responses are identical (number of trailer axles, fifth axle spacing, and longitudinal bending moment of inertia). Both responses are governed by the degree of local load concentration and the rigidity of the bridge structure. The wheelbase 5 determines the positional relationship of the axle group relative to the control section, while the longitudinal bending moment of inertia reflects the fundamental geometric characteristics of the bridge structure. Together, these factors dictate the extent of amplification for the most unfavorable effect. (3) The combination of key features differs for the negative bending moment response, with the critical factors being bridge span length, total bridge length, and the number of trailer axles. This is because negative bending moments primarily occur in the support regions of continuous beams, and their magnitude is fundamentally governed by the span length and total bridge length. Longer spans result in a wider influence range and higher peak negative moments. Simultaneously, the axle count contributes to the total load, and together these factors predominantly drive the amplification effect for negative bending moments. (4) Axle weight exhibits a relatively minor influence on the mixed traffic load effect ratio. This is attributable to the concentrated distribution of axle weight for both tractors and trailers within the input dataset, which already approaches the regulatory load limits. This reflects the impact of mandatory specifications on transportation practices. Should policies change, the axle load distribution of ROVs would also change. Consequently, the prediction model requires periodic updates in accordance with vehicle management policies and in-vehicle load levels.

## 8. Conclusion

This study presents a machine learning-driven framework for rapid assessment of bridge responses under mixed traffic of ROVs and ordinary vehicles. By integrating high-fidelity finite element simulations, influence surface-based loading, and ensemble learning algorithms, the method effectively bridges the gap between computational efficiency and accuracy in evaluating spatial load coupling effects. The key findings are as follows:

(1) This study systematically established and quantified the load effect amplification factor (*ξ*) for mixed traffic scenarios, demonstrating that bridge response is influenced not only by total vehicle weight, but more significantly by axle configuration, bridge type, and span length.
(2) An influence surface-based moving load analysis approach was implemented, enabling efficient batch simulation of over 2,000 real ROVs across 17 bridge types, significantly reducing computational cost compared to traditional dynamic simulation methods.
(3) A tree-based ensemble model (XGBoost) was successfully trained to predict *ξ* with high accuracy (*R*² > 0.98), providing a reliable and rapid tool for augmenting conventional single-vehicle assessment methods in engineering practice. The SHAP analysis indicates that under current vehicle load limit standards, axle loads have a relatively minor impact on the mixed traffic effect ratio. The number of trailer axles emerges as a key feature across all three mixed response types. Furthermore, trailer axle spacing and the longitudinal bending moment of inertia significantly influence the amplification factors for positive bending moment and shear force. For negative bending moment, bridge span length and total bridge length also rank among the most influential factors.
(4) The proposed framework supports real-time and batch safety evaluations of bridges under mixed traffic, offering a scalable and intelligent solution for permit decision-making and bridge management.
(5) The parameters of ROVs may vary in different regions (for instance, the transportation vehicles for wind turbine blades in western provinces of China are longer and have more axles). Therefore, in practical applications, it is recommended to use local or recent licensing data to fine-tune or retrain the model. Furthermore, when calculating the load effect of ROVs on highway bridges in this study, it is assumed that the ROVs pass the bridges at a relatively low speed (5–10 km/h), and the unevenness of the bridge surface conforms to the technical condition under normal maintenance conditions. If the actual driving speed of the ROVs significantly increases (for example, exceeding 20 km/h), or if the bridge surface is severely uneven, the dynamic influence may further increase, a more detailed vehicle-bridge coupling analysis should be adopted.

Future work may extend the method to include dynamic load effects, more diverse traffic scenarios, and network-level bridge management systems, further enhancing its applicability in intelligent transportation infrastructure management.

## Supporting information

S1 File The complete dataset of this study. This file includes all the parameters of over 2,000 ROVs, structural parameters of 17 bridges, and the corresponding calculated values of amplification factors.
(RAR)

## References

[1] Yuan Y, Han W, Xu X, Wang J, Sun J. Permit checking of overloaded customized transport vehicle based on serviceability limit state reliability of concrete bridges. Adv Struct Eng. 2021;24:884–96. doi: 10.1177/1369433220972451

[2] Casas JR, Aparicio AC. Computer-based bridge management system for permit vehicle routing. Comput Civ Infrastruct Eng. 2001;16:444–54. doi: 10.1111/0885-9507.00246

[3] Liang J, Tan C, Yan L, Zhou J, Yin G, Yang K. Interaction-aware trajectory prediction for safe motion planning in autonomous driving: a transformer-transfer learning approach. IEEE Trans Intell Transport Syst. 2025;26(10):17080–95. doi: 10.1109/tits.2025.3588228

[4] Liang J, Yang K, Tan C, Wang J, Yin G. Enhancing high-speed cruising performance of autonomous vehicles through integrated deep reinforcement learning framework. IEEE Trans Intell Transport Syst. 2025;26(1):835–48. doi: 10.1109/tits.2024.3488519

[5] Li Y, Zhan Y, Liang M, Zhang Y, Liang J. UPTM-LLM: Large language models-powered urban pedestrian travel modes recognition for intelligent transportation system. Appl Soft Comput. 2026;186:113999. doi: 10.1016/j.asoc.2025.113999

[6] Choi HH, Lim JK, Seo JW, Jung PK. An overweight permit analysis system for bridge management. KSCE J Civ Eng. 2006;10(2):123–9. doi: 10.1007/bf02823930

[7] Correia JR, Arruda MRT, Branco FA. Structural assessment of reinforced-concrete arch underpasses subjected to vehicular overloads. J Perform Constr Facil. 2014;28:321–9. doi: 10.1061/(asce)cf.1943-5509.0000410

[8] Liu B, Han W, Wang T, Feng Y, Wang J, Xu K. Analytical method for overturning risk assessment of curved girder bridges with single-column piers under heavy-duty vehicles. Structures. 2023;57:105084. doi: 10.1016/j.istruc.2023.105084

[9] Liu B, Han W, Zhao W, Wang T, Liu X, Wang J, et al. Safety assessment of short- and medium-span bridges with mixed traffic of customized transport vehicles and ordinary vehicle flow considering non-stationary factors. China J Highway Transp. 2024;37(8):111–24. doi: 10.19721/j.cnki.1001-7372.2024.08.010

[10] Wang J, Liu B, Yuan S, Wang T, Han W. Safety evaluation of special-purpose vehicle crossing small and medium span bridge under unclosed traffic condition. J Zhejiang Univ Eng Sci. 2025;59(5):1092–102.

[11] Technical Specification for Safety Passage Assessment of Highway Oversize and Overweight Transport (JTG/T 2213-2023). Beijing: China Communications Press; 2023.

[12] Liu B, Wang Y, Hu P, Yuan Q. Impact coefficient and reliability of mid-span continuous beam bridge under action of extra heavy vehicle with low speed. J Cent South Univ. 2015;22(4):1510–20. doi: 10.1007/s11771-015-2668-6

[13] Lenner R, Sýkora M. Partial factors for loads due to special vehicles on road bridges. Eng Struct. 2016;106:137–46. doi: 10.1016/j.engstruct.2015.10.024

[14] Yan J, Deng L, He X. Optimal transverse position for overweight trucks to cross simply supported multi-girder bridges. Adv Struct Eng. 2018;21(9):1251–61. doi: 10.1177/1369433217737116

[15] Deng L, Yan W. Vehicle weight limits and overload permit checking considering the cumulative fatigue damage of bridges. J Bridg Eng. 2018;23:1–8. doi: 10.1061/(asce)be.1943-5592.0001267

[16] Gao W, Yuan Y, Huang P, Xu X, Wang J, Han W. Safety assessment of steel strand stay cable under customized transport vehicle load considering strength degradation. China J Highway Transp. 2020;33(8):169–81. doi: 10.19721/j.cnki.1001-7372.2020.08.017

[17] Zhang J, Kong X, OBrien EJ. Computer vision-based weight identification and stability evaluation of exceptional transport vehicles. Eng Struct. 2023;294:116773. doi: 10.1016/j.engstruct.2023.116773

[18] Huang P, Wang J, Han W, Yuan Y. Study on impact factors of small- and medium-span bridges under the special-purpose vehicle load. Structures. 2022;43:606–20. doi: 10.1016/j.istruc.2022.06.077

[19] Hua Z, Chengjun T, OBrien EJ, Bin Z, Nasim U, Hongjie G. Developing digital twins to characterize bridge behavior using measurements taken under random traffic. J Bridg Eng. 2022;27:4021101. doi: 10.1061/(ASCE)BE.1943-5592.0001814

[20] Huang P, Wang J, Xu X, Yang G, Chen S, Yuan Y, et al. Improved multi-lane traffic flow simulation based on weigh-in-motion data. Meas J Int Meas Confed. 2022;188:110408. doi: 10.1016/j.measurement.2021.110408

[21] General specifications for design of highway bridges and culverts. Beijing: China Communications Press; 1989.

[22] General specifications for design of highway bridges and culverts (JTG D60-2004). Beijing: China Communications Press; 2004.

[23] General specifications for design of highway bridges and culverts (JTG D60-2015). Beijing: China Communications Press; 2015.

[24] Zhou J, Wu W, Caprani CC, Tan Z, Wei B, Zhang J. A hybrid virtual–real traffic simulation approach to reproducing the spatiotemporal distribution of bridge loads. Comput-Aided Civ Infrastruct Eng. 2024;39(11):1699–723. doi: 10.1111/mice.13154

[25] Zhou J, Zheng Y, Tan Z, Wei B, Zhou X, Ruan X. Feasibility study of bridge load testing using ongoing traffic through rapid estimation of traffic load effects for network-level highway bridges assisted by site-specific ETC data. J Civil Struct Health Monit. 2025;15(6):1793–813. doi: 10.1007/s13349-025-00911-3

[26] Han W, Yuan Y, Xie Q, Gao G, Chen X, Xu K. Load effect, safety assessment, and traffic strategy of multigirder bridges under lateral eccentric customized transport vehicle. J Perform Constr Facil. 2019;33:4018110. doi: 10.1061/(ASCE)CF.1943-5509.0001257

[27] Han W, Liu X, Gao G, Xie Q, Yuan Y. Site-specific extra-heavy truck load characteristics and bridge safety assessment. J Aerosp Eng. 2018;31:4018098. doi: 10.1061/(ASCE)AS.1943-5525.0000917

[28] Luk SH. Machine learning-based methods for the seismic damage classification of RC buildings. Buildings. 2025;15(14):2395. doi: 10.3390/buildings15142395

[29] Arachchilage CB, Zhao J, Dushyantha N, Liu WV. A transformer-based machine learning model for optimizing the design of cementitious mixtures with mine tailings as supplementary cementitious materials. Cem Concr Compos. 2026;165:106363. doi: 10.1016/j.cemconcomp.2025.106363

[30] Xie Y, Yao G, Yuan Z. Study on maximum temperature under multi-factor influence of tunnel fire based on machine learning. Buildings. 2025;15(18):3401. doi: 10.3390/buildings15183401

[31] Wang J, Lu J, Zhang J, Sun J, Yang G. A β-VAE-stacking ensemble prediction model for fatigue life of reinforced concrete beams. Structures. 2025;79:109616. doi: 10.1016/j.istruc.2025.109616

